# Trace Metal and Metalloid Profiles in Hair Samples from Children in the Oil-Producing Region of Kazakhstan

**DOI:** 10.3390/toxics13070522

**Published:** 2025-06-21

**Authors:** Gulnara Batyrova, Victoria Kononets, Gulmira Umarova, Gulaim Taskozhina, Yeskendir Umarov, Zhamilya Issanguzhina, Khatimya Kudabayeva, Rabbil Batyrov

**Affiliations:** 1 Department of Clinical Laboratory Diagnostics, West Kazakhstan Marat Ospanov Medical University, Aktobe 030019, Kazakhstan; batyrovagulnara77@gmail.com (G.B.); taskozhina.gulayim@gmail.com (G.T.); 2 Department of Natural Sciences, West Kazakhstan Marat Ospanov Medical University, Aktobe 030019, Kazakhstan; eskendir.um@gmail.com (Y.U.); areskz123@gmail.com (R.B.); 3 Department of Evidence-Based Medicine and Scientific Management, West Kazakhstan Marat Ospanov Medical University, Aktobe 030019, Kazakhstan; 4 Department of Children Disease No. 2, West Kazakhstan Marat Ospanov Medical University, 68 Maresyev Street, Aktobe 030019, Kazakhstan; gamilia0452@gmail.com; 5 Department of Internal Diseases 1, West Kazakhstan Marat Ospanov Medical University, Aktobe 030019, Kazakhstan; h.kudabaeva@zkmu.kz

**Keywords:** human hair, children, toxic trace elements, oil production, Western Kazakhstan

## Abstract

Toxic elements are considered a significant threat to public health in oil-producing countries. Western Kazakhstan is experiencing serious environmental problems due to the development of the oil and gas industry. This study aimed to assess the concentrations of toxic trace elements—aluminum (Al), arsenic (As), beryllium (Be), cadmium (Cd), mercury (Hg), and lead (Pb)—in the hair of children residing in Kazakhstan’s oil and gas-producing region, and to evaluate the relationship between the concentration of toxic elements and the remoteness of their residence from oil and gas fields. A cross-sectional analysis was conducted involving 1595 school-aged children. Element levels in hair samples were quantified using inductively coupled plasma mass spectrometry (ICP-MS). The association between trace element concentrations and residential distance from oil and gas fields was examined across three distance-based groups and further analyzed through multiple linear regression. The highest concentration of Al = 4.824 μg/g and Hg = 0.096 μg/g was found in the hair of children living close to oil and gas fields (0–16 km). A decrease in levels of Al (−0.072 (CI: −0.109; −0.036)) and Hg (−0.293 (CI: −0.343; −0.243)) is associated with increasing distance from oil and gas fields. As, Cd, and Pb had the lowest median concentrations in the hair of children living near oil and gas fields (0.030, 0.010, and 0.122 µg/g, respectively). There is a tendency for levels of As, Cd, and Pb to increase with distance from the fields (0.064 (CI: 0.039; 0.089), 0.093 (CI: 0.045; 0.141), and 0.244 (CI: 0.202; 0.287), respectively). Our findings indicate the need for biomonitoring of toxic elements to determine long-term temporal trends in the influence of toxic trace elements on the health of the child population of Western Kazakhstan.

## 1. Introduction

Population growth, rapid urbanization, and industrialization have led to severe environmental pollution by toxic trace elements originating from human activities across various global regions, either through direct input or indirect influence [1]. This issue is particularly relevant for Kazakhstan, where intensive industrial development has resulted in significant air and soil pollution, especially in urban areas [2]. However, both urban and rural areas of Western Kazakhstan face serious environmental challenges due to the expansion of the oil and gas industry over the past decades. Kazakhstan is the largest regional oil producer after Russia [3].

Heavy metals are considered a significant threat to public health in oil-producing countries [4]. Toxic trace elements (TEs) such as Pb, Al, Mn, Ni, and Cd can harmfully affect various organs in the human body. Therefore, monitoring and controlling their toxicity is essential to protect vulnerable populations [4]. Various scientific reports demonstrated a clear association between air pollution exposure and health outcomes, as quantified through dose-response modeling. The effects of air pollution on human health are complex, and it is difficult to quantify the effect of a particular pollutant [3]. However, toxic trace elements have adverse effects with long-term exposure, even at low doses [5]. Living in regions contaminated with toxic trace elements increases the risk of adverse public health effects. Monitoring of trace elements in the body, especially toxic trace elements, is necessary to protect the health of children, who are particularly vulnerable to environmental pollutants [6]. Children and adolescents should be the target population for environmental and public health research [1]. Children’s development and health can be affected by toxic exposure to heavy metals [7]. Arsenic (As), mercury (Hg), lead (Pb), and cadmium (Cd) have been identified as the most hazardous elements affecting health [8]. As, Pb and Hg have well-documented adverse neurological effects. The toxic elements aluminum (Al), As, Cd, Hg, and Pb have been shown in several studies to have adverse effects on child development [9,10,11]. Children are more vulnerable to environmental pollutants than adults. They have a less developed blood-brain barrier, which allows xenobiotics, including toxic metals, to enter and accumulate in the nervous system [12,13]. Toxic elements can enter children’s bodies through ingestion of food, beverages, or contaminated airborne particles, among other routes [14]. Children breathe more air, drink more water, and eat more food per unit of body weight than adults [15]. Infants and children are more likely to be exposed to contaminated soil than adults because of their behavioral patterns [16] and higher rates of absorption through the gastrointestinal tract [17]. Their habit of putting their hands in their mouths and the fact that they live and play close to the ground make them more vulnerable than adults [18]. It is, therefore, important to remember that exposure to even low doses of toxic elements can have adverse effects on children’s bodies [19].

Human hair is a useful tissue sample for non-invasive environmental studies [20]. Measuring the concentration of toxic and potentially toxic elements in scalp hair can be a useful method for exposure studies and pollution assessment. Hair is one of the most attractive biomaterials because it is easy to collect, store, transport, and prepare samples for analysis [21]. Hair reflects long-term exposure better than blood [20]. According to some researchers, the use of human hair is controversial because of various confounding factors that may affect the presence of trace elements in hair [1]. Factors influencing the results included external atmospheric contamination of hair, the use of cosmetics, hair dyeing, and the use of metal instruments when taking samples for research [22]. According to Molina-Villalba et al., trace element levels in hair do not correlate with levels found in urine, with the exception of total mercury content [6]. However, the use of methodology and strict inclusion criteria can facilitate the use of human hair as a screening tool in environmental biomonitoring studies [23]. Hair samples are ideal for the analysis of healthy populations in population studies of interest for public health issues [14]. In recent years, there has been an increase in the number of studies that have measured the content of several elements in hair in both unpolluted and polluted areas [20,24,25,26,27,28], with some of these studies focusing exclusively on child populations.

In many countries, biomonitoring systems have been established to assess the level of population exposure by determining concentrations of both essential and toxic elements in biological samples [27,29,30,31,32,33]. Of particular significance is the focus on the pediatric population, given their heightened vulnerability to environmental influences during critical stages of development [34]. The global prevalence and significance of biomonitoring and assessing the effects on children’s health and development, particularly in relation to toxic elements, have been identified as a pressing concern and a priority for research in many regions [14].

Despite extensive research on environmental contamination in various regions related to industrial and technical impacts, as well as in the area of former and active military ranges, there is an absence of national biomonitoring systems in Kazakhstan, and there is a paucity of regular tracking of data on the level of toxic trace elements in adults and children. Studies measuring the content of toxic trace elements in the hair of the population of different regions of Kazakhstan are few. A substantial proportion of these studies focuses on the bioelement status of the population in the western region of Kazakhstan [35,36,37]. Our previous research delineated the elemental status by the content of toxic elements in the hair of the adult population of the oil-producing region.

We present data on the levels of toxic trace elements in scalp hair samples from children in Western Kazakhstan living at different distances from oil and gas extraction and processing facilities to test whether they are valuable in reflecting residential toxic trace element contamination.

The aim of this study was to measure the concentration of the toxic trace elements Al, As, Be, Cd, Hg, and Pb in the hair of children from the oil and gas province of Kazakhstan and to evaluate the relationship between the concentration of toxic elements and the remoteness of their residence from oil and gas fields. We also compared our results with other biomonitoring studies of toxic elements in children’s hair described in the literature.

## 2. Materials and Methods

### 2.1. Study Population

A cross-sectional study of toxic trace elements in hair (Al, As, Be, Cd, Hg, Pb) was conducted in 2023–2024 in three oblasts of Western Kazakhstan, the largest industrial region of the country. The territory of Western Kazakhstan is located in the arid climatic zone. The landscape is represented by desert, semi-desert, and, in the north of the region, steppe. The lack of transport infrastructure limits communication in the rural areas of the region and makes access to medical services problematic for the inhabitants. In addition, the low income level of the rural population reduces the diversity of the diet. A problem of the arid climate region is inadequate access to good quality drinking water. Terrestrial and underground freshwater resources are often contaminated by industrial and oil production products, and in Mangistau oblast, they are practically non-existent, and the population consumes desalinated sea water.

Trace elements were measured in the scalp hair of healthy children within the 5–18 age range. The study included 1595 school-age children permanently residing in Aktobe, Mangistau, and Atyrau oblasts by cluster sampling method (Table 1).

To calculate the sample, we used official data on the number of the general population by age from 5 to 18 years old in three regions of Western Kazakhstan [38].

Inclusion criteria: age—school-aged children of both genders (from 5 years to 18 years old), belonging to any ethnic group, permanently residing in the territory of Western Kazakhstan; written informed consent of parents/legal representatives before inclusion in the study. Exclusion criteria: insufficient hair length to obtain a useful sample, acute inflammatory or infectious diseases; severe concomitant somatic diseases; hereditary diseases and congenital malformations; vegetarian diet; use of vitamin and mineral supplements; presence of metal implants; undergoing any hair care procedures within 3 months prior to sampling (hair coloring, products applied to the hair and scalp for therapeutic purposes), smoking. Schools were selected in each administrative unit of all included in the study oblasts of Western Kazakhstan. Subjects were randomly selected on site from those who were present on the day of sample collection and from those whose parents provided authorized written permission to participate in the study. A total of 1595 samples were collected from 30 schools, one school per district. Weight and height were measured, and age and sex were recorded. Parents of students were asked to respond to a questionnaire regarding social and demographic data [39].

**Figure 1:**
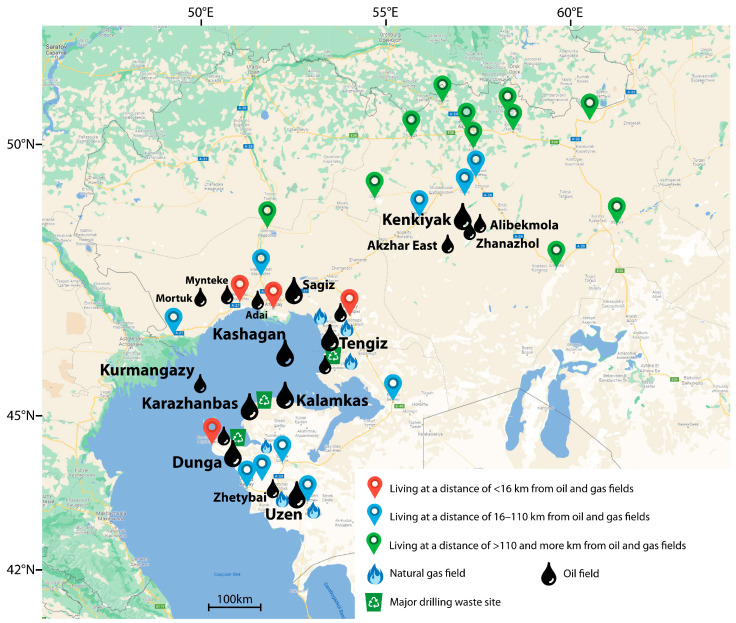
Map of the surveyed areas.

Trace element levels in the hair of children residing at varying distances from oil and gas fields were compared across three zones: less than 16 km, between 16 and 110 km, and more than 110 km. Distances within 16 km are considered the zone where emissions from oil and gas facilities may impact human health. [40]. When comparing trace element levels for the results of residents living beyond 110 km, we assumed that they were relatively uncontaminated [41]. The geographic distribution of sampling sites was georeferenced and analyzed using QGIS (version QGIS 3.34.11), an open-source Geographic Information System. Precise coordinates for each sampling location were collected using government-provided data, ensuring accurate spatial referencing. All spatial data were projected to an appropriate coordinate reference system (EPSG:3857—WGS84) to enable distance calculations. Euclidean (straight-line) distances from each sampling site to the nearest oil field were calculated using the “Distance to Nearest Hub” and “Distance Matrix” tools in QGIS (Figure 1).

### 2.2. Ethical Issues

The study received approval from the local ethics committee of Marat Ospanov West Kazakhstan Medical University (meeting No. 7.02 of 29 September 2022). The aims and objectives of the study, design, and methods of research were carried out in accordance with the Constitution of the Republic of Kazakhstan, “Code of the Republic of Kazakhstan on the health of the people and the health care system”, principles of the Helsinki Declaration and subsequent amendments. Before parents answered the questionnaire and hair samples were taken from their children, the aims and methods of the study were explained to them, and informed consent was obtained in writing.

### 2.3. Data Collection

Hair samples were collected by cutting with clean stainless steel scissors from 3–5 areas of the occipital region, ensuring a minimum total weight of 0.1 g. For elemental analysis, the proximal segments of the strands, measuring 3–4 cm in length, were utilized. The samples were then sealed in labeled plastic envelopes and stored at room temperature until analysis.

Hair elemental composition studies were performed by inductively coupled plasma mass spectrometry (ICP-MS) on an Agilent 8900 ICP-MS mass spectrometer (Agilent Technologies, Santa Clara, CA, USA), with an automatic pipette SPS4 (Agilent Technologies, USA).

Hair samples were subjected to sample preparation by washing and microwave decomposition. The hair strands were washed with acetone, then rinsed three times with deionized water and air-dried at 60 °C. The sample (0.05 g sample weight) was decomposed with HNO3 (Fluka #02650 Sigma-Aldrich, Co., Burlington, MA, USA) in a Berghof SW-4 DAP-40 microwave system (Berghof Products + Instruments GmbH, Eningen, Germany) and diluted with distilled deionized water to a concentration of 0.5–1% HNO_3_ before direct injection into the ICP-MS system.

The ICP-MS system is prepared for operation according to factory specifications and calibrated by external calibration to calibration multi-element standards. Tuning Solution 10 µg/L Ce, Co, Li, Tl, Y in 2% acid (Agilent Technologies, USA) is used for daily evaluation of the mass spectrometer sensitivity. The level of oxides and doubly charged ions does not exceed <1.5%.

Standards containing the full spectrum of the elements to be determined in concentrations of 0.5, 1, 5, 10, and 5000 µg/L are prepared before starting work from the Universal Data Acquisition Standards Kit reference solutions (#N9306225, PerkinElmer Inc., Waltham, MA, USA) by dilution in distilled deionized water acidified with 1% HNO_3_. To account for the incomplete matching of sample matrices and calibration solutions for acidity and viscosity, the assay uses an online internal standardization for rhodium isotope 103. An internal standard containing 10 µg/L Rh is prepared from a rhodium reference standard (#N9300167, PerkinElmer Inc.) on a matrix containing 8% butanol-1 (#1. 00988, Merck KGaA, Darmstadt, Germany), 0.8% Triton X-100 (Sigma #T9284 Sigma-Aldrich, Co., Burlington, MA, USA), 0.02% TMAH (#20932, Alfa-Aesar, Ward Hill, MA 01835 USA) and 0.02% EDTAcid (Sigma#431788 Sigma-Aldrich, Co.). Sample collection was performed using an SPS4 autosampler, and sample introduction was performed using an ISIS 3 system (both Agilent Technologies). To eliminate polyatomic overlays, an ammonia-helium mixture is used as the reaction cell gas.

### 2.4. Statistical Methods

Kolmogorov–Smirnov and Shapiro–Wilk criteria were applied to assess the normality of the distribution of quantitative variables.

Key descriptive statistics, including the arithmetic mean (AM), geometric mean (GM), median (Me), and minimum and maximum values, along with the 2.5th, 25th, 50th, 75th, and 97.5th percentiles, were calculated for data characterization.

Qualitative variables are reported as absolute values and percentages. The concentrations of all toxic elements in scalp hair were not normally distributed. Non-parametric tests, including the Mann–Whitney U test and Kruskal-Wallis test, were employed to assess statistically significant differences between quantitative variables. The relationship between the concentrations of hair elements and the considered factors was studied using the Spearman correlation test.

Multiple linear regression analysis was applied to assess the relationship between concentrations of toxic trace elements in the hair (dependent variable) and the distance of the settlement from oil and gas production sites (independent variable). Prior to analysis, data were log-transformed using natural logarithm Ln(X) to meet the assumption of the regression model. The distribution of log-transformed variables was assessed using descriptive statistics and graphical methods. To control for potential confounding, the models included the following covariates, based on self-reported questionnaire data: age (continuous), gender (two categories), body mass index (BMI, continuous), place of residence (two categories), and tobacco smoking (two categories). A significance threshold *p* < 0.05 was adopted for hypothesis testing, with results reported at a 95% confidence interval

Data analysis was conducted using SPSS version 25 (SPSS Inc., Chicago, IL, USA) and Statistica version 10 (StatSoft Inc., Tulsa, OK, USA).

## 3. Results

### 3.1. Content of Toxic Elements in the Scalp Hair of Children from Western Kazakhstan

The children lived at a distance of 0.2 to 376 km from the oil and gas extraction area. The mean age of the study participants was 11 (5; 18) years. 764 (47.9%) of the study participants were boys. Approximately equal numbers of the study population lived in urban areas, 805 (50.5%) and 790 (49.5%) in rural areas (Table 1).

Descriptive statistics of the levels of toxic elements (Al, As, Be, Cd, Hg, Pb) in the children’s hair in the oil and gas province of Kazakhstan are presented in Table 2. The unit of measurement is a microgram of element per gram of dry hair samples. The order of concentration of elements in hair with decreasing content: Al > Pb > Hg > As > Cd > Be. The distribution of values for all toxic elements in the described sample was asymmetric, with a pronounced shift to the left, forming curves of leptokurtic type. This conditioned the analysis on the basis of median and percentiles.

### 3.2. Associations of Children’s Hair Toxic Element Level with Age and Gender

Differences in hair trace element concentrations were observed at significantly higher levels in boys for most of the toxic elements (Al, As, Be, Cd, and Pb) (*p* < 0.001). Hg concentrations did not differ significantly by gender (Table S1, Supplementary Materials). The relationship between age and toxic trace element concentrations was also investigated (Table S1, Supplementary Materials). The age of the children was considered as a numerical variable, and the degree of correlation between age and toxic trace element concentrations in children’s hair was measured using Spearman’s Rho coefficient. The correlation coefficients ranged from −0.320 (for Pb) to 0.080 (for Hg). The age of the children was significantly related to hair concentrations of all toxic elements except beryllium; younger children had higher levels of toxic elements in their hair than older children. However, for all elements that showed either positive or negative correlations with age, the correlation coefficients were below 0.5, indicating weak associations.

### 3.3. Association Between Distance to Oil and Gas Sites and Hair Levels of Toxic Trace Elements

Table 3 shows the concentrations of Al, As, Be, Cd, Hg, and Pb in the hair of children living permanently at different distances from oil and gas fields and refineries in Western Kazakhstan. Significant differences in hair levels of all toxic trace elements were found between the three groups of study participants. The highest concentrations of Al and Hg were found in the hair of study participants living close (0–16 km) to oil and gas fields. Hg levels in the hair of children living close to oil and gas fields (0–16 km) were more than 2 times higher than those of children from communities farthest from the fields (more than 110 km). Be concentrations were highest in the hair of children living 16–110 km from oil and gas fields. However, As, Cd, and Pb levels in the hair of children living near oil fields were significantly lower than those of children from more distant settlements.

Multiple regression analysis was used to further investigate the effect of the distance of children’s homes from oil fields and oil and gas refineries on the levels of toxic elements in hair. We examined how the association between concentrations of toxic trace elements in children’s hair changed with distance, taking into account the influence of variables such as age, sex, BMI, smoking, and urban/rural residence. The results are summarized in Table 4. To illustrate how this table should be understood, we use the example of As. In model 0, the regression coefficient of As, 0.066 (95% CI 0.040; 0.092), means that the natural logarithm of the concentration of As in the hair of children living in the oil-producing region of Kazakhstan increases by the indicated value with each additional 100 km. A distance of 100 km was selected across all models to enhance the significance of the linear regression coefficients. The adjusted coefficients indicate the variation in arsenic concentration in hair relative to distance, accounting for different sets of covariates: age in Model A; age and gender in Model B; age, gender, and BMI in Model C; age, gender, BMI, and parental smoking in Model D; and finally, age, gender, BMI, parental smoking, and residential setting (urban or rural) in Model E (see Table 4).

Multiple linear regression analysis revealed a significant inverse association between distance from oil and gas sites and hair concentrations of aluminum and mercury, with regression coefficients of −0.072 (95% CI: −0.109; −0.036) and −0.293 (95% CI: −0.343; −0.243), respectively. These findings suggest that children residing farther from extraction and processing areas had lower levels of Al and Hg in their hair. Conversely, other toxic elements such as arsenic, cadmium, and lead exhibited a positive association, with concentrations increasing as residential distance from oil and gas activities increased (Table 4).

## 4. Discussion

This study is the first to determine hair concentrations of toxic elements of children of the oil- and gas-bearing region of Kazakhstan. We have previously described the concentrations of trace elements, including toxic elements, in the adult population of this region [36,37]. Table 5 shows the concentrations of toxic elements in the hair of children from different countries, including polluted and unpolluted regions.

In this study, we evaluated the impact of several independent variables on the levels of toxic elements such as aluminum, arsenic, beryllium, cadmium, mercury, and lead in children’s hair, including age, gender, BMI, distance from home to oil and gas extraction and refining facilities, location of home (rural/urban), and parental smoking.

Differences across age and gender groups have been described in the majority of population studies on toxic trace element concentrations in children’s hair [6,7,24,46,54,55]. Despite significant differences in the results of these studies, they are united by the conclusion that gender is a significant variable that must be carefully accounted for when interpreting metal concentrations in human hair [46]. In our study, all toxic elements except Hg showed clear significant gender differences. As shown in Table S1 (Supplementary Materials), the concentrations of Al, As, Be, Cd, and Pb were much higher in the hair of boys. Our results are analogous to studies that found higher concentrations of the toxic elements Pb [27,45], Cd [45], and Hg [27] in the hair of male children. However, a number of studies show clearly opposite results, where the concentrations of Al [7,44], As [6], Cd [7,24], and Be [7] are higher in the hair of girls. Gender differences in hair element concentrations may be due to several factors (such as height, physiology, sex hormones, and lifestyle), resulting in different responses of boys and girls to chemical exposure [7,23,56]. Despite the conflicting results, they all support the importance of gender in the ability to accumulate toxic elements in hair, and the differences in results may be due to the different age groups of children included in the study (Table 5).

Age is an equally important factor, as shown by the correlation of the hair’s toxic elements content with the age of the children studied (Table S1). All toxic trace elements except mercury showed a negative correlation with the age of the children. The decrease in the concentration of such elements as Al, As, and Be in hair with age is confirmed by the data of our previous study in the adult population of the oil-producing region of Kazakhstan [36].

Numerous studies confirm that the elemental profile in the hair of both adult and child populations depends on the local environmental conditions formed by a complex of anthropogenic and natural factors [27,57,58]. This is true both for polluted regions [20,26,27,42,43], including oil-producing regions [36,59,60], and for populations living in unpolluted regions [14,25,49,50,51].

The influence of oil and gas production on environmental pollution by toxic trace elements is a recognized fact [59,60]. The peculiarities of the local geochemical provinces in the oil-producing areas and the technological differences in the process of oil and gas extraction and the utilization of wastes, especially wastewater, cause the difference in the spectrum of pollutant metals in the oil-producing industrial regions. An increase in the content of aluminum [61], arsenic [62,63], cadmium [64], mercury [6], beryllium [6,65], and lead [66,67] in the soil in the area of oil production and processing complexes has been noted.

In our study, the highest concentrations of aluminum (Al) and mercury (Hg) in hair were observed among children residing within 0–16 km of oil and gas extraction sites (Table 3), which is confirmed by the data of linear regression model construction (Table 4). It is noteworthy that we obtained similar results in a previous study on the determination of toxic elements in the population of adults living in this region [36].

Studies on the profile of toxic elements in the hair of children living in contaminated areas have usually focused on determining the concentration of As, Cd, Hg, and Pb as the priority and most toxic [6,68,69,70]. Aluminum and beryllium have been studied less frequently. However, several investigations have detected higher levels of Al in the hair samples of children living both in contaminated areas [43,44] and in areas where residence is associated with problems of water supply and access to good quality drinking water [7].

Aluminum, the most widely distributed metal in the Earth’s crust [71], is not essential for human metabolism and can be toxic to the body [72]. The primary sources of aluminum in nature are rocks, with a smaller contribution from surface and underground water, as well as soil. [73]. The transfer of Al from the lithosphere to the biosphere is primarily a consequence of human-induced processes, either directly or indirectly [74]. The primary method of exposure to aluminum in humans is through ingestion, primarily through the consumption of food, particularly grains, vegetables, processed foods, water, and medications containing aluminum, such as antacids, buffer analgesics, anti-diarrheal agents, or ulcer-healing drugs [72]. Inhalation and dermal contact may also make small contributions to the daily human exposure to aluminum [71]. However, respirable aluminum is mainly available to occupationally exposed groups [72] and cannot affect the pediatric population. When considering aluminum levels in the pediatric population, it is important to note that aluminum is a potent adjuvant commonly employed in vaccine formulations [75]. Research has revealed that the levels of aluminum in infant formula are considerably higher than in breast milk [76]. However, throughout Western Kazakhstan, there are no differences between regions in terms of vaccination coverage among children and the vaccines and infant formula used, which, like vaccines, are produced outside Kazakhstan. Thus, the most likely source of elevated aluminum levels in children living near oil and gas fields is food (cereals, vegetables) and water. It is important to recognize that aluminum in food originates not only from its natural presence due to environmental exposure and food additives but also from contact with aluminum-based materials used in packaging and cooking processes [77]. Aluminum absorption is determined not only by its content in food and water but also by other factors, such as the pH of food, Al compound, and the presence of other substances. Iron deficiency in the diet plays a particularly important role in increasing aluminum absorption. Iron deficiency anemia is one of the health problems in Kazakhstan, especially for vulnerable groups such as children and pregnant women. The prevalence of iron deficiency anemia in Kazakhstan is higher in rural areas, as well as in regions with significant industrial pollution [78].

Elevated aluminum levels in children have a detrimental effect on their health, especially in cases of concomitant kidney disease [75], and lead to bone disease due to impaired phosphate absorption [71], nervous system and developmental problems, and difficulties in socialization [79].

Currently, there are no specific, generally accepted recommendations regarding aluminum (Al) content in hair based on health protection principles.

**Figure 2:**
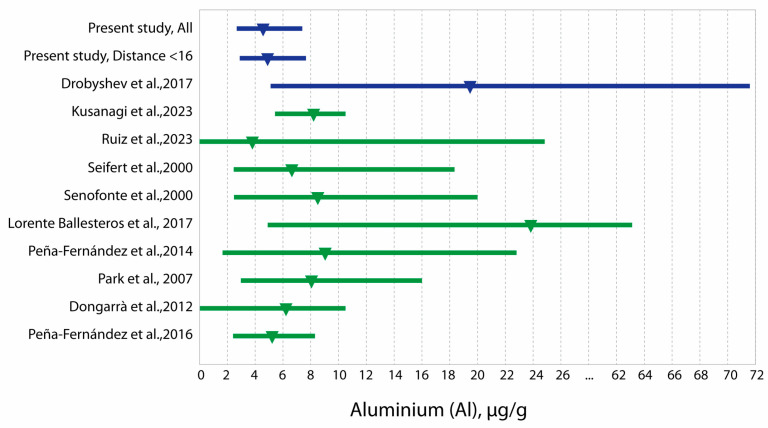
Hair aluminum concentrations in children from Western Kazakhstan compared with literature data (medians and ranges) [1,14,22,24,33,42,43,46,47,48]. The dark blue color indicates polluted areas, and the green color indicates non-polluted areas.

Analysis of aluminum levels in children’s hair in various populations shows that studies conducted in contaminated areas and in exposed populations represent a smaller proportion of the total number of papers (Table 5). Nevertheless, high concentrations of hair aluminum have been observed in exposed and unexposed populations of children (Figure 2). A comparable pattern was evident in the elemental profiles of adults living in contaminated and uncontaminated areas [36].

As one of the most dangerous toxic metals, mercury presents serious health risks to children. Mercury is a worldwide contaminant that accumulates in the environment, primarily through the food chain in aquatic ecosystems, posing a significant health hazard to children. Mercury can exist in various chemical forms, including elemental (or metallic), inorganic, and organic (methylmercury and ethylmercury) [80]. Upon being released into the environment, mercury is rapidly converted by microorganisms into organic compounds. These compounds have the tendency to bioaccumulate and biomagnify within the bodies of animals [81]. Organic mercury, the most common form of which is methylmercury, poses a significant threat to human health [80]. The level of mercury in hair is associated with a decrease in neurobehavioral indicators and changes in brain structure in young people [82]. The sources of mercury exposure encompass mining, industrial processes, dietary intake, religious practices, traditional medicine, cosmetics, dental fillings, and the management of waste [83]. Children are exposed through air, water, food, and soil [80]. In developing countries, children are frequently exposed to elevated levels of mercury in comparison to their counterparts residing in developed countries. This discrepancy can be attributed to a number of factors, including the prevalence of mercury-intensive industrial processes and consumer products, the absence of stringent environmental regulations, and the limitation of food options [84]. Numerous investigations emphasize the significance of toxic metals, such as mercury, as environmental contributors to the development of autism, particularly in nations with limited resources. Children with autism exhibited notably elevated mercury levels in their hair [85,86,87].

The Joint Food and Agriculture Organization of the United Nations and WHO (FAO/WHO) Expert Committee on Food Additives (JECFA) has established a guidance value of 2.3 μg/g for Hg in hair [88]. The U.S. Environmental Protection Agency (EPA) has established a more stringent reference dose (RfD) for chronic oral exposure to methylmercury (MeHg), set at 0.1 µg/kg body weight per day, to protect against developmental neuropsychological deficits. This corresponds to a mercury concentration of 1 μg/g in hair for children and women of reproductive age [89,90].

**Figure 3:**
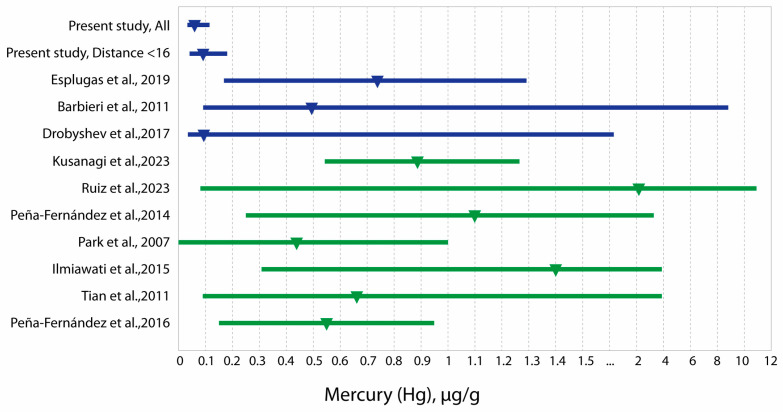
Hair mercury concentrations in children from Western Kazakhstan compared with literature data (medians and ranges) [1,14,22,26,33,41,42,48,50,51]. The dark blue color indicates polluted areas, and the green color indicates non-polluted areas.

Elevated concentrations of mercury in soil have been observed in regions with oil production and developed petrochemical industries [59]. Elevated mercury concentrations in the hair of pediatric populations have been reported in Bolivia in a region exposed to polymetallic mining waste [42] and in Spain in schoolchildren located near facilities involved in hazardous waste incineration [27]. In our previous study of an adult population in an oil-producing region of Kazakhstan, mercury also showed significantly higher concentrations in the hair of the population living in the areas closest to oil and gas extraction and processing sites [36]. However, hair mercury concentrations in adults in this region are almost three times higher than in children (Table 5). High hair mercury concentrations have been reported not only in the hair of children living in contaminated areas but also in unexposed child populations [1,14,34,51,52] (Table 5) (Figure 3).

Regions whose gastronomy is rich in fish and seafood are currently of particular concern because of their ability to bioaccumulate toxic elements, including mercury [14,91,92]. Studies conducted in geographical regions where fish and seafood are consumed in abundance, such as Japan [34,51], the Mediterranean [1,5,14,93], and the Canadian Arctic [52], demonstrate a correlation between dietary fish and seafood consumption and the concentration of mercury in children’s hair. The diet of the inhabitants of the Western Kazakhstan region is rich in fish compared to populations in other regions of Kazakhstan due to the proximity of the Caspian Sea and certain problems with traditional livestock production (droughts in the desert region and lack of fodder for cattle). However, the ecological state of the region should also be taken into account. The waters of the Caspian Sea and adjacent areas are polluted due to active oil production in the region, including drilling in the coastal zone and on the shelf. The proximity to West Kazakhstan of a significant number of Russian oil and gas production facilities in the Astrakhan and Orenburg regions, as well as the presence of military weapons testing sites (Orenburg region, Totskoye) and military launch sites for space equipment and intercontinental ballistic missiles (Orenburg region, Yasnoye, and Astrakhan region, Kapustin Yar) should also be taken into account.

At first sight, our data on the increase of arsenic, cadmium, and lead concentrations in the hair of children living here are somewhat contradictory (Table 3 and Table 4). It should be taken into account that in the areas far from the fields, there are large cities in the region where the chemical industry and ferrous and non-ferrous metallurgy enterprises—active sources of pollution of the living environment—are located. In addition, it is necessary to take into account not only anthropogenic sources of pollution but also geological sources of metal input into the environment. However, when compared with data from other exposed and unexposed populations, even the highest levels of some toxic elements in our study are much lower than the corresponding levels found in these studies. For example, hair arsenic levels among children in exposed populations were (µg/g) 2.038 (0.975; 3.699) in Russia (Karabash, copper and polymetallic ore mining region) [26], 0.79 (0.10; 3.30) in Bolivia (Altiplano, polymetallic ore mining region) [42], in unexposed populations 0.53 (0.14; 2.9) in Bangladesh [50], 0.12 (<0.05; 0.26) in Spain (Madrid) [25], 0.39 (0.11; 1.24) in Bolivia (urban population) [42], whereas in our study it was 0.032 (0.023; 0.044), which is several times lower than the reported values. Most studies in exposed and unexposed pediatric populations also show higher levels of cadmium and lead in hair, often several times higher than our values (Table 3).

Studies of polluted areas differ in their methodological approach and, above all, in the nature, origin, and intensity of the pollution, which makes it difficult to compare data from different sources. In the area where oil and gas extraction takes place, it is necessary to take into account the peculiarities of the technologies used, the extraction methods, and the composition of the drilling mud, wastewater, and sludge polluting the area. It is also necessary to take into account the differences in the elemental composition of soils in biogeochemical provinces where different populations live. Studies comparing the elemental profiles of adults and children of the same population or of populations living in neighboring regions, either simultaneously or sequentially, are of particular importance. In the present study, a statistically confirmed trend of decreasing aluminum and mercury concentrations in the hair of the child population with increasing distance from oil and gas extraction and refining sites was observed, which is consistent with similar data from an earlier study in an adult population in Western Kazakhstan. Nevertheless, the relatively elevated concentrations of arsenic, cadmium, and lead observed in populations residing at greater distances from oil and gas fields suggest an overall ecological imbalance in the region, highlighting the need for enhanced biomonitoring and strengthened chemical safety interventions.

This study has some limitations.

1. As a cross-sectional study, the present research does not permit the assessment of changes over time.
2. The contribution of food consumption to the elemental composition of hair has not been assessed, including the impact of fish and cereal consumption, which may influence cadmium and mercury levels in hair.
3. The content of toxic elements in soil, water, and air was not assessed.
4. This study did not include an assessment of toxic element concentrations in other biological matrices like blood or urine.
5. The capacity of oil and gas fields and, accordingly, the intensity of pollution produced by them were not taken into account.

## 5. Conclusions

Significantly higher hair concentrations of the toxic trace elements Al, As, Be, Cd, and Pb were observed in boys compared to girls. The age of the children had a significant negative correlation with the concentrations of all toxic elements in the hair, except for Hg, for which a significant positive correlation was described. There are substantial variations in the concentrations of toxic metals such as aluminum, arsenic, cadmium, mercury, and lead in hair between children of Western Kazakhstan residing at varying distances from oil and gas extraction facilities (0–16 km, 17–110 km, more than 110 km). The highest concentration of Al and Hg was found in the hair of children living close to oil and gas fields (0–16 km). A decrease in Al and Hg levels is associated with increasing distance from oil and gas fields, and factors such as age, gender, BMI, and smoking status do not alter this relationship. Concentrations of As, Cd, and Pb in hair increase with distance from oil and gas production sites. The data obtained by us indicate the need for biomonitoring of toxic elements to determine long-term temporal trends in the impact of toxic trace elements on the health of the child population of Western Kazakhstan.

A multifaceted approach is required to address the contamination of the population in the oil-producing region of Western Kazakhstan with toxic trace elements Al and Hg. Firstly, additional research is needed to fill the existing gaps in the data on the true magnitude of exposure from various sources, including the determination of contamination of water, air, soil, and food products with toxic metals in the region. A set of public health measures is needed, including well-developed rules and recommendations on safe levels of Al and Hg in air, water, soil, or food, and policies implemented to reduce the impact of these toxic elements on the child population. Allocated resources, including financial support, equipment, and skilled personnel, should accompany this.

## Figures and Tables

**Table 1:** Anthropometric and socio-demographic characteristics of children and parents.

Studied Population Characteristics	^1^ N	^2^ %	^3^ Median (Min–Max)
Total	1595	100	
Gender	1595	100	
Boys	764	47.9	
Girls	831	52.1	
Age (years)	1595	100	11 (5–18)
5–9	619	38.8	
11–18	976	61.2	
Height, cm	1595	100	144 (84–189)
Weight, kg	1595	100	37 (14–124)
Body mass index, kg/m^2^	1595	100	17.36 (10.77–40.32)
Oblasts	1595	100	
Aktobe	573	35.9	
Mangistau	410	25.7	
Atyrau	612	38.4	
Place of residence	1595	100	
Urban	805	50.5	
Rural	790	49.5	
Distance of place of residence to oil and gas fields, km	1595	100	
0–16	524	32.9	
17–110	579	36.3	
110 km and more	492	30.8	
Passive smoking	1595	100	
Yes	729	45.7	
No	866	54.3	
Monthly income, tenge	1595	100	
Up to 50 thousand	119	7.5	
50–100 thousand	479	30.0	
100–150 thousand	245	15.4	
150–200 thousand	240	15.0	
200–250 thousand	389	24.4	
250 thousand and above	123	7.7	

^1^ N = data number. ^2^ N% = data number percentage. ^3^ Median (min–max) = median (minimum–maximum range).

**Table 2:** Toxic element levels (µg/g) in the hair of children living in the Western Kazakhstan region (*n* = 1595).

Element	AM	GM	Me	Min	Percentile				Max
					2.5th	25th	75th	97.5th	
Al	6.45	4.41	4.40	0.309	0.904	2.59	7.29	24.36	277.05
As	0.039	0.033	0.032	0.003	0.012	0.023	0.044	0.106	0.984
Be	0.002	0.001	0.001	0.000005	0.0002	0.0004	0.001	0.003	1.05
Cd	0.022	0.011	0.011	0.0001	0.001	0.005	0.024	0.111	0.878
Hg	0.105	0.060	0.058	0.003	0.008	0.028	0.122	0.466	2.46
Pb	0.324	0.174	0.170	0.013	0.025	0.079	0.362	1.57	13.60

AM—arithmetic mean; GM—geometric mean.

**Table 3:** Comparative analysis of toxic trace element concentrations (µg/g) in the hair of children from Western Kazakhstan across three groups based on residential distance from oil and gas production and processing sites.

Element	Remoteness < 16 km (*n* = 524)				Remoteness 16–110 km (*n* = 579)				Remoteness > 110 km (*n* = 492)				*p* K-W
	AM	GM	Me (q25; q75)	P2.5; P97.5	AM	GM	Me (q25; q75),	P2.5; P97.5	AM	GM	Me (q25; q75),	P2.5; P97.5	
Al	6.154	4.791	4.824 ^c^ (2.975; 7.771)	(1.250; 19.414)	7.061	4.617	4.517 ^c^* (2.656; 7.346)	(1.076; 28.907)	6.061	3.822	3.704 ^a,b^* (2.126; 7.009)	(0.540; 25.350)	0.001
As	0.033	0.030	0.030 ^b,c^ (0.022; 0.040)	(0.012; 0.077)	0.042	0.034	0.032 ^a^ (0.022; 0.040)	(0.013; 0.132)	0.041	0.034	0.033 ^a^ (0.024; 0.047)	(0.013; 0.116)	<0.001
Be	0.0006	0.0010	0.0005 ^b,c^ (0.0003; 0.0008)	(0.0001; 0.0020)	0.003	0.001	0.0010 ^a,c^ (0.0005; 0.0016)	(0.0001; 0.0030)	0.0008	0.0007	0.0007 ^a,b^ (0.0004; 0.0012)	(0.0001; 0.0022)	<0.001
Cd	0.020	0.010	0.010 ^b,c^** (0.005; 0.020)	(0.001; 0.124)	0.023	0.010	0.013 ^a^ (0.005; 0.020)	(0.001; 0.119)	0.022	0.012	0.012 ^a^** (0.005; 0.026)	(0.001; 0.100)	<0.001
Hg	0.137	0.090	0.096 ^b,c^ (0.047; 0.173)	(0.013; 0.474)	0.105	0.053	0.051 ^a,c^*** (0.024; 0.111)	(0.008; 0.609)	0.071	0.044	0.043 ^a,b^*** (0.025; 0.077)	(0.005; 0.355)	<0.001
Pb	0.260	0.132	0.122 ^b,c^ (0.063; 0.269)	(0.021; 0.904)	0.325	0.178	0.174 ^a,c^ (0.082; 0.337)	(0.029; 1.811)	0.392	0.226	0.229 ^a,b^ (0.108; 0.467)	(0.033; 1.921)	<0.001

AM—arithmetic mean; GM—geometric mean. Significance values were adjusted for multiple tests using Bonferroni correction. Post hoc comparisons: differences at the level of *p* < 0.001 for a, b, and c; *p* = 0.004 for b* and c*; *p* = 0.005 for a** and c**; *p* = 0.041 for b*** and c***; a—<16 km; b—16–110 km; c—>110 km.

**Table 4:** Evaluation of crude and adjusted differences in hair levels of toxic trace elements: findings from multiple regression analysis.

Element	Model 0	95% CI	*p*	Model A	95% CI	*p*	Model B	95% CI	*p*	Model C	95% CI	*p*	Model D	95% CI	*p*	Model E	95% CI	*p*
Al	−0.062	(−0.103; −0.022)	0.003	−0.052	(−0.088; −0.015)	0.006	−0.044	(−0.080; −0.008)	0.017	−0.044	(−0.080; −0.008)	0.017	−0.072	(−0.108; −0.036)	<0.001	−0.072	(−0.109; −0.036)	<0.001
As	0.066	(0.040;0.092)	<0.001	0.071	(0.045; 0.096)	<0.001	0.080	(0.055; 0.104)	<0.001	0.079	(0.055; 0.103)	<0.001	0.064	(0.040; 0.089)	<0.001	0.064	(0.039; 0.089)	<0.001
Be	0.038	(−0.003; 0.078)	0.071	0.040	(−0.001; 0.081)	0.055	0.040	(−0.001; 0.081)	0.054	0.039	(−0.002; 0.080)	0.060	0.031	(−0.011; 0.073)	0.144	0.030	(−0.011; 0.072)	0.155
Cd	0.073	(0.018;0.128)	0.009	0.089	(0.041; 0.137)	<0.001	0.102	(0.055; 0.149)	<0.001	0.101	(0.055; 0.148)	<0.001	0.092	(0.044; 0.140)	<0.001	0.093	(0.045; 0.141)	<0.001
Hg	−0.245	(−0.294; −0.195)	<0.001	−0.244	(−0.293; −0.194)	<0.001	−0.249	(−0.299; −0.200)	<0.001	−0.250	(−0.299; −0.200)	<0.001	−0.294	(−0.344; −0.244)	<0.001	−0.293	(−0.343; −0.243)	<0.001
Pb	0.220	(0.169; 0.271)	<0.001	0.235	(0.191; 0.279)	<0.001	0.253	(0.212; 0.295)	<0.001	0.252	(0.211; −0.293)	<0.001	0.244	(0.202; −0.288)	<0.001	0.244	(0.202; 0.287)	<0.001

**Table 5:** Summary of published data on concentrations of toxic trace elements (μg/g) in hair in different populations.

Sample Type & Location	Age (Years)	*n*		Al Median (Range)	As Median (Range)	Be Median (Range)	Cd Median (Range)	Hg Median (Range)	Pb Median (Range)	References
Present study, total sample	5–18	1595	Median P25; P75	4.40 (2.59; 7.29)	0.032 (0.023; 0.044)	0.001 (0.0004; 0.001)	0.011 (0.005; 0.024)	0.058 (0.028; 0.122)	0.170 (0.079; 0.362)	
Present study, living at a distance of 0–16 km from oil fields	5–18	524	Median P25; P75	4.824 (2.975; 7.771)	0.030 (0.022; 0.040)	0.0005 (0.0003; 0.0008)	0.010 (0.005; 0.020)	0.096 (0.047; 0.173)	0.122 (0.063; 0.269	
West Kazakhstan	adults	850	Median P25; P75	4.080 (1.007; 33.586)	0.030 (0.004; 0.116)	0.0004 (0.0001; 0.0026)	0.010 (0.001; 0.141)	0.145 (0.019; 1.035)	0.181 (0.032; 2.651)	Batyrova et al., 2022 [36]
Spain, Catalonia	10–13	94	Mean ± SD	-	-	-	0.04 ±0.05	0.73 ± 0.56	1.44 ± 1.89	Esplugas et al., 2019 [27]
Russia, Karabash	14.7 ± 1.1 years	46	Median P25; P75	-	2.038 (0.975–3.699)	-	0.118 (0.055–0.210)	-	5.44 (3.57–13.98)	Skalny et al., 2018 [26]
Bolivia (mining area)	7–9	60	GM P5–P95	-	0.79 0.10–3.30	-	0.07 0.00–2.03	0.49 0.09–8.44	14.08 3.24–65.4	Barbieri et al., 2011 [42]
Russia	7.6 ± 1.3	82	Median Min–max	19.5 5.1–71.7	0.020 <0.003–0.330		0.11 0.01–1.01	0.09 <0.03–2.23	2.48 0.33–28.0	Drobyshev et al., 2017 [43]
Italy, Sicily, Industrial area	11–13	60	Median P10; P90	4.1 (2.0; 8.3)	0.038 (0.002; 0.119)	-	0.077 (0.001; 0.148)	-	1.58 (0.59; 3.29)	Dongarrà et al., 2012 [44]
Italy, the active volcanic area of Mt. Etna	11–13	376	Median P10–P90	4.90 2.08; 8.38	0.03 0.004; 0.06	-	0.01 0.002; 0.04	-	0.58 0.14; 1.89	Varrica et al., 2014 [20]
Poland	8–15	158	Me (Min; Max)	-	-	-	0.83 (0.08;3.73)	-	6.74 (0.57;36.14)	Chłopicka et al., 1998 [45]
Sicily	11–14	963	Median P25; P75	5.0 (0.01; 10.6)	0.03 (0.0003; 0.17)	-	0.01 (0.0003; 0.18)	-	0.63 (0.03; 4.0)	Tamburo et al., 2016 [46]
Non-polluted area										
Italy, Sicily, Rural area	11–13	47	Median P10; P90	4.2 (2.5; 8.1)	0.04 (0.02; 0.07)	-	0.037 (0.008; 0.057)	-	0.70 (0.42; 1.78)	Dongarrà et al., 2012 [44]
Japan	3–6	118	Median P25; P75	8.17 (5.41; 10.78)	-	-	0.001 (0.0007; 0.0011)	0.88 (0.54; 1.26)	0.96 (0.62; 1.49)	Kusanagi et al., 2023 [34]
Spain	3–12	419	Median P5; P95	5.98 (0.00; 24.78)	0.020 (0.002; 0.068)	0.000 (0.000; 0.004)	0.025 (0.002; 0.146)	2.09 (0.08; 10.98)	1.17 (0.08; 7.39)	Ruiz et al., 2023 [14]
Africa, Ethiopia	0–18	81	GM P25; P75 mg kg^−1^	1 (<DL; 42)	0.04 (<DL; 0.09)	0.008 (0.004; 0.027)	0.10 (0.06; 0.21)	0.056 (0.026; 0.111)	3.1 (1.7; 5.4)	Astolfi et al., 2020 [7]
Germany	6–14	711	GM P10; P90	6.57 (2.4; 18.3)	-	-	0.048 (0.01; 0.19)	-	1.02 (0.3; 3.5)	Seifert et al., 2000 [47]
Italy, Rome	3–14	412	Median P5; P95	8.45 (2.4; 20.0)	0.06 (0.14; 0.24)	-	0.14 (0.04; 0.61)	-	5.60 (1.0; 19.8)	Senofonte et al., 2000 [48]
Spain, Madrid	0–18	648	Mean P5; P95	23.8 4.9; 63.2	0.12 <0.05; 0.26	-	0.029 0.004; 0.079	-	1.23 0.17; 4.28	Llorente Ballesteros et al., 2017 [25]
Spain, Alcalá de Henares	6–9	117	AM P5; P95	9.05 1.71; 22.76	ND	ND	0.52 0.17; 0.93	1.10 0.25; 3.35	1.48 0.25; 3.47	Peña-Fernández et al., 2014 [23]
South Korea	3–6	655	Median P5; P95	8.08 (3; 16)	0.11 (0.05–0.20)	-	0.07 (0.01–0.20)	0.43 (0–1)	1.43 (<3)	Park et al., 2007 [49]
Bolivia	7–9	71	GM P5–P95	-	0.39 0.11–1.24	-	0.08 0.03–0.19	0.15 0.05–0.50	2.32 0.33–10.19	Barbieri et al., 2011 [42]
Bangladesh	9–10	207	Median P5; P95	-	0.53 (0.14–2.9)	-	29 (0.76–150)	-	1.6 (0.50–6.4)	Skröder et al., 2017 [50]
Japan	9–10	229	Median Min–Max	--	--	-	-	1.40 0.31–3.96	-	Ilmiawati et al., 2015 [51]
Canada	3–5	361	GM P10–P90	-	-	-	-	0.66 0.09; 3.96	-	Tian et al., 2011 [52]
Italy, Sicily	11–13	131	Median P10; P90	6.09 (0.01; 10.49)	0.0003 (0.0003; 0.014)	-	0.03 (0.01; 0.11)	-	0.78 (0.37; 1.84)	Dongarrà et al., 2012 [44]
Spain	13–16	96	M ± S.D	5.34 ± 2.96	ND	ND	0.11 ± 0.14	0.55 ± 0.40	0.70 ± 0.52	Peña-Fernández et al., 2016 [1]
Southern Brazil	12–18	126	Median P25; P75	-	0.006 (0.001; 0.02)	-	0.003 (0.000; 0.02)	-	0.1 (0.009; 0.4)	Carneiro et al., 2011 [53]

## Data Availability

Data are available from the corresponding author upon request.

## Supplementary Materials

The following supporting information can be downloaded at: https://www.mdpi.com/article/10.3390/toxics13070522/s1, Table S1: Age and gender influence on toxic element levels (µg/g) in Western Kazakhstan children’s hair.

## Abbreviations

The following abbreviations are used in this manuscript:

ICP-MS	inductively coupled plasma mass spectrometry
BMI	body mass index
AM	arithmetic mean;
GM	geometric mean
