# Supplementary material for: Trace Metal and Metalloid Profiles in Hair Samples from Children in the Oil-Producing Region of Kazakhstan
Source: Toxics. 2025 Jun 21;13(7):522. doi: 10.3390/toxics13070522 (PMC12298664; doi:10.3390/toxics13070522)
Supplement: Supplementary file 1 [file toxics-13-00522-s001.zip › Table S1.pdf]

Supplementary Materials

Table S1. Age and gender influence on toxic element levels (µg/g) in Western Kazakhstan children's hair.

Element	Rho Spearman	p-values	Males (n=764)									Females (n=831)									p M-W U test
							Percentile									Percentile				Max	
			AM	GM	Me	Min	2.5th	25th	75th	97.5th	Max	AM	GM	Me	Min	2.5th	25th	75th	97.5th		
Al	-0,181	<0.001	8,935	6,404	6,018	0,336	1,732	3,911	9,945	34,952	277,047	4,174	3,128	3,184	0,309	0,666	1,905	5,159	13,222	44,568	<0.001
As	-0,288	<0.001	0,047	0,038	0,037	0,003	0,015	0,026	0,052	0,132	0,984	0,032	0,028	0,028	0,004	0,011	0,021	0,037	0,077	0,153	<0.001
Be	-0,001	0,961	0,0010	0,0007	0,0008	0,0000	0,0000	0,0004	0,001	0,003	0,026	0,0021	0,0006	0,0006	0,0000	0,0000	0,0003	0,001	0,003	1,048	<0.001
Cd	-0,222	<0.001	0,033	0,020	0,020	0,001	0,003	0,010	0,037	0,167	0,878	0,011	0,007	0,007	0,0000	0,001	0,003	0,013	0,049	0,196	<0.001
Hg	0,080	0.001	0,115	0,062	0,060	0,003	0,009	0,029	0,129	0,518	2,461	0,096	0,057	0,057	0,003	0,008	0,028	0,115	0,435	1,124	0,299
Pb	-0,320	<0.001	0,503	0,299	0,286	0,026	0,049	0,151	0,564	2,036	13,596	0,160	0,106	0,104	0,013	0,022	0,055	0,197	0,588	3,070	<0.001
